# Comparison of the characteristics of mesenchymal stem cells obtained from prostate tumors and from bone marrow cultured in conditioned medium

**DOI:** 10.3892/etm.2012.642

**Published:** 2012-07-20

**Authors:** GUANXIONG DING, JIALIANG SHAO, QIANG DING, ZUJUN FANG, ZHONG WU, JIANFENG XU, PENG GAO

**Affiliations:** 1 Department of Urology, Huashan Hospital, Fudan University, Shanghai, P.R. China;; 2 Department of Urology, Wake Forest University School of Medicine, Winston-Salem, NC, USA

**Keywords:** prostate cancer, mesenchymal stem cells, RM-1

## Abstract

Prostate cancer (PCa) is the most common type of cancer worldwide. Mesenchymal stem cells (MSCs) can also be utilized as ‘tumor stromal cells’, which are associated with invasive and metastatic malignant tumor cells. Our study aimed to investigate MSCs in prostate tumors and normal MSCs and evaluate their differential characteristics. Normal MSCs (BMMSCs) were isolated from the femur and tibia of normal mice; prostate tumor MSCs (PCa-MSCs) were obtained from prostate tumors implanted in mice. These two types of MSCs were induced to differentiate into adipocytes, bone cells and chondrocytes. Growth curves were used to analyze the growth ability of PCa-MSCs and BMMSCs. Tritium-labeled thymidine (3H-TdR) was used to evaluate cell proliferation of RM-1 stimulated by MSCs. The time taken for PCa-MSCs to reach 90% confluence was markedly shorter than that of BMMSCs (8–10 vs. 12–14 days). The differentiation ability of PCa-MSCs was similar to that described in previous reports. The growth ability of PCa-MSCs was significantly higher than that of BMMSCs. The proliferative activity of PCa-MSCs was also higher than that of BMMSCs. Our data showed that PCa-MSCs exhibit identical characteristics when compared with those of MSCs. Additionally, their proliferative activity and growth ability were significantly higher when compared with these values in BMMSCs, which appear to have an intrinsic, cell-specific capacity to localize to PCa. The possible role of PCa-MSCs in the process of PCa development requires further clarification.

## Introduction

Prostate cancer (PCa) is the most common type of cancer worldwide, and still ranks as the leading cause of death among urological malignancies. One in six men will be diagnosed with PCa during their lifetime and approximately 217,000 new cases were diagnosed in the US in 2010 (1). PCa progression involves tumor cell proliferation and infiltration into surrounding tissue and induction of various adaptive pathophysiological and pathomorphological processes in tissues that become involved in the developing tumor stroma. However, the mechanism involved in the development of PCa is still unclear.

Within the bone marrow stroma there exists a subset of nonhematopoietic cells referred to as mesenchymal stem cells (MSCs) (2–4). These cells can be expanded *ex vivo* and induced *in vitro* or *in vivo* to terminally differentiate into osteogenic, adipogenic, chondrogenic, and myogenic lineages under appropriate conditions. In addition, MSCs migrate to sites of injury and inflammation and tumors (5,6). Phenotypically, MSCs are identified by the absence of the CD34 and CD45 hematopoietic cell markers and are positive for Thy-1 (CD90), endoglin (CD105), vascular cell adhesion molecule-1 (VCAM-1/CD106), SH2 and SH3. MSCs express major histocompatibility complex (MHC) class I but do not express MHC class II, B7-1, B7-2, CD40, or CD40L molecules (7).

MSCs can be utilized as ‘tumor stromal cells’, targeting invasive and metastatic malignant tumor cells (8). Djouad *et al* (9) also found that MSCs were associated with side effects related to systemic immunosuppression favoring tumor growth *in vivo*. It is therefore important to investigate the factors related to the *in vivo* promotion of tumor growth by MSCs and explore the safety of clinical applications of MSCs. As the microenvironment of PCa is similar to that of injured/stressed tissue (10,11), it was hypothesized that PCa may provide a conducive environment for the grafting of exogenously administered MSCs (12).

Therefore, in the present study, we aimed to investigate the characteristics of MSCs obtained from bone marrow or prostate tumors. Additionally, we investigated whether the proliferation of grafted MSCs in the developing tumors is capable of generating a significant fraction of tumor stroma.

## Materials and methods

### 

#### Animal subjects

Male mice (nu/nu) were purchased from the Animal Production Area of the Fudan University Cancer Research Center. All animal manipulations were carried out in accordance with Fudan University guidelines under approved protocols. Male four-week-old BALB/c mice (n=20) were maintained and bred under specific pathogen-free conditions, and were divided into the various experimental groups.

#### Cell lines and animal model

RM-1 cells were purchased from the Institute of Cell Biology, Shanghai, China and cultured in DMEM supplemented with 10% fetal bovine serum, 100 U/ ml penicillin, 100 g/ml streptomycin at 37°C in a humidified 5% CO_2_ incubator. BALB/c mice (n=12) inoculated subcutaneously with RM-1 cells were used as a model of prostate cancer whereas the control group comprised 8 BALB/c mice injected with physiological saline. The time and the efficiency of cancer formation were measured.

#### Mesenchymal stem cell isolation and culture

The methods described by Peister *et al* were used (13). Briefly, MSCs were extracted from the femur, tibia and humerus of normal mice (BMMSCs) under axenic conditions by washing with PBS and filtration through a 200-mesh sieve net. Prostate tumors were obtained within 15 days following injection. Tumor tissue isolated from the mice with prostate cancer was cut into 3 mm^3^ pieces. Prostate tumor MSCs (PCa-MSCs) were obtained after filtration through a 200-mesh sieve net and centrifugation. CD105 cells separated using magnetic beads were cultured in DMEM-LG medium containing 10% fetal bovine serum.

#### In vitro efficacy experiments of MSCs

MSCs are progenitors of skeletal tissue components such as bone, cartilage and adipocytes. To ascertain the *in vitro* differentiation ability of MSCs isolated from bone marrow stroma and prostate tumor we induced differentiation using a previously described method (14–16).

#### Growth ability of the BMMSCs and PCa-MSCs

The growth ability of the two types of MSCs (BMMSCs vs PCa-MSCs) was compared using growth curves. The RM-1 cell concentration was adjusted to 1×10^7^/ml with RPMI-1640. The RM-1 cells were grown in 96-well culture plates (Nunc Inc.) with 1x10^6^/well density, to which different concentrations (1:1, 1:2, 1:3, 1:4 and 1:5) of BMMSCs or PCa-MSCs were added. The 96-well culture plates were cultured in DMEM/F12 medium (37°C, 5% CO_2_). The culture was terminated prior to 12–16 h by adding 100 μl (0.5–1 μCi) tritium labeled thymidine (3H-TdR) to each well. After the end of the culture, the cells were collected on glass fiber filter paper for natural drying. Scintillation counting (cpm) values were determined each minute using a beta liquid scintillation counter. This experiment was also performed with the control group (PBS).

#### Statistical analysis

All data are analyzed using the SigmaStat statistical software (Jandel Scientific, San Rafael, CA, USA) and S SigmaPlot (SPSS Inc. Chicago, IL, USA). P<0.05 was considered to indicate a statistically significant difference.

## Results

### Cell culture

**Figure 1:**
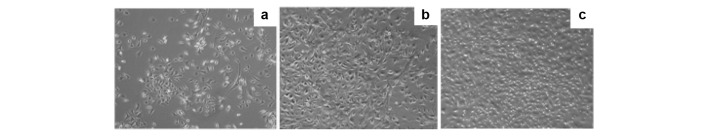
Characterization of isolated PCa-MSCs from the prostate tumors: (a) 48 hours after plating and (b) 7 days after plating as detected using microscopy (magnification, ×100). (c) The third generation of PCa-MSCs as detected using microscopy (magnification, ×100).

**Figure 2:**
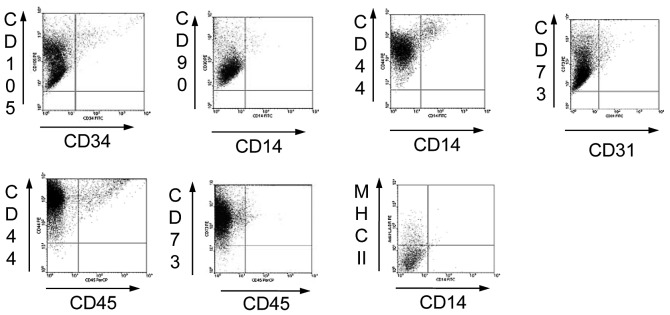
The P3 PCa-MSCs exhibited high expression of CD44, CD73, CD90 and CD105, but were negative for CD14, CD34, CD45 and MHC-II. The PCa-MSCs were 95% homogeneous.

The results of the BMMSC culture were similar to those of previous reports (17). We therefore focused on the results for the PCa-MSCs culture. Fig. 1a demonstrates the cell growth status 48 h after primary vaccination in the culture medium. We used microscopy to observe the extent of cell growth and the number of colonies, which were fusiform. The time required for 90% confluence of the PCa-MSCs was markedly shorter than that of the BMMSCs (Fig. 1b; 8–10 days vs. 12–14 days). PCa-MSCs were cultured, often with a mixture of various cells, past 2–3 generations and became uniform in morphology (Fig. 1c). As detected by flow cytometry, the P3 PCa-MSCs exhibited high expression of CD44, CD73, CD90 and CD105, but were negative for CD14, CD34, CD45 and MHC-II. The PCa-MSCs were 95% homogeneous (Fig. 2).

### Differentiation of PCa-MSCs

#### PCa-MSCs were induced to differentiate into adipocytes (Oil red O)

**Figure 3:**
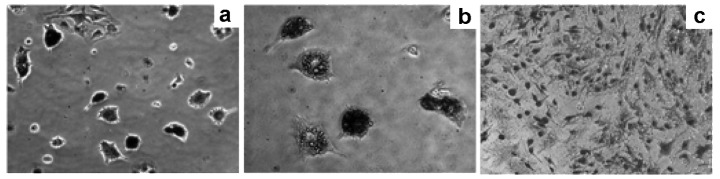
PCa-MSCs were induced to differentiate into adipocytes (Oil red O). (a) The PCa-MSCs exhibiting a long spindle-shape gradually become oval or round at 14 days as detected under microscopy (magnification, ×100). (b) Intracytoplasmic refractive bright circular lipid droplets were noted at 14 days under microscopy (magnification, ×100). (c) The normal control cytoplasm was not stained by Oil red O.

When induced by adipogenic medium, the PCa-MSCs exhibiting a long spindle-shaped form gradually became oval or round (Fig. 3a), with intracytoplasmic refractive bright circular lipid droplets (Fig. 3b). Lipid droplets exhibited a brick-red color after staining with Oil red O while the normal control cytoplasm was not stained (Fig. 3c).

#### PCa-MSCs were induced to differentiate into bone cells (alkaline phosphatase)

**Figure 4:**
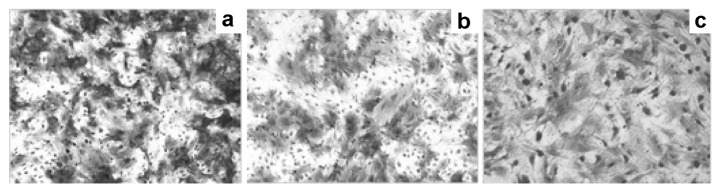
PCa-MSCs were induced to differentiate into bone cells (alkaline phosphatase). (a) The cell morphology of PCa-MSCs changed from a spindle-shaped to a flat-shape at 10 days as detected under microscopy (magnification, ×40). (b) More red alkaline grain-containing acid enzyme-positive granules in the cytoplasm at 10 days were noted under microscopy (magnification, ×100). (c) In the normal controls, alkaline phosphatase expression was almost negative as noted under microscopy (magnification, ×100).

With osteogenic induction, the cell morphology of PCa-MSCs changed from a spindle-shaped to a flat-shaped morphology (Fig. 4a) and cells were observed to be alkaline phosphatase-positive after 10 days (more red alkaline grain-containing acid enzyme-positive granules in the cytoplasm, Fig. 4b), whereas in the normal controls, alkaline phosphatase expression was almost negative (Fig. 4c).

#### PCa-MSCs were induced to differentiate into bone cells (alizarin red)

**Figure 5:**
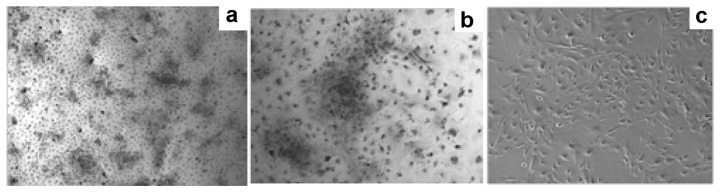
PCa-MSCs were induced to differentiate into bone cells (alizarin red): (a) with 0.1% alizarin red staining at 14 days under microscopy (magnification, ×40). (b) Visible orange-red nodules and a clear boundary of the mineralized nodules were noted at 14 days as detected under microscopy (magnification, ×100). (c) The normal control cells showed negativity to alizarin red under microscopy (magnification, ×100).

The PCa-MSCs exposed to osteogenic induction with 0.1% alizarin red staining exhibited visible orange-red nodules and a clear boundary of the mineralized nodules after 14 days (Fig. 5a and b), whereas normal control cells showed negativity to alizarin red staining (Fig. 5c).

#### PCa-MSCs were induced to differentiate into chondrocytes (toluidine blue)

**Figure 6:**
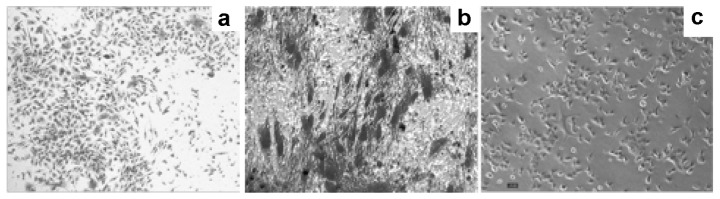
PCa-MSCs were induced to differentiate into chondrocytes (toluidine blue). (a) The PCa-MSCs continued to proliferate to form multiple cell nodules; cells were polygonal or round shape at 10 days as detected under microscopy (magnification, ×40). (b) The cells exhibited blue metachromasia in the cytoplasm when stained with toluidine blue at 10 days after induction as detected under microscopy (magnification, ×100). (c) The normal control cells were negative as detected under microscopy (magnification, ×100).

After chondrogenic induction, the PCa-MSCs continued to proliferate to form multiple cell nodules, in which cells were polygonal or round in shape (Fig. 6a). We observed that the cells exhibited blue metachromasia in the cytoplasm stained with toluidine blue 10 days after induction (Fig. 6b). The normal control cells were negative (Fig. 6c).

#### Proliferative activity and growth ability of BMMSCs and PCa-MSCs

**Figure 7:**
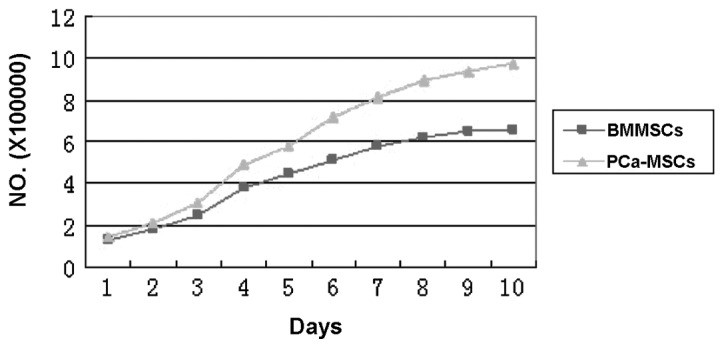
Growth curves of the PCa-MSCs and BMMSCs.

**Figure 8:**
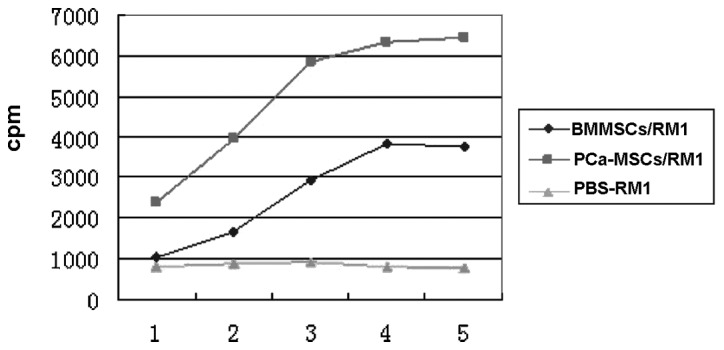
RM1 cell proliferation experiments evaluated by 3H-TdR.

We found that the growth ability of PCa-MSCs was markedly higher than that of BMMSCs. The growth curve of these two cell types is shown in Fig. 7. Based on the RM-1 cell proliferation experiments we found that the proliferative activity of PCa-MSCs was also higher than that of the BMMSCs (Fig. 8).

## Discussion

In this study, we provide evidence that MSCs home to subcutaneously implanted mouse prostate tumors, which was similar to the results of previous studies, in which cells were observed in the lung and liver. Our methods for PCa-MSC isolation and culture were effective, since the cells showed a phenotype of CD34^−^/CD45^−^ [not hematopoietic cells (18,19)], CD44^+^/CD73^+^/CD105^+^/CD90^+^ [mesenchymal stromal cell and stem cell markers (20–22)] and CD14^−^/MHC-II^−^ [not endothelial progenitor cells (21–23)]. The isolation of PCa-MSCs from prostate tumors is an important aspect of our study. In fact, to our knowledge, this is the first demonstration of PCa-MSCs obtained from prostate tumors implanted in mice. Alternatively, human PCa, similar to other cancers, requires the elaboration of mesodermal elements, specifically endothelial cells and pericytes. It has been suggested that MSCs are a main source of pericytes within the bone marrow stroma (24,25); thus, MSCs may integrate into prostate cancer to contribute to the mesenchymal elements of the tumor. MSCs may localize to the tumor under physiological conditions to assist with tissue repair. This results in a microenvironment conducive to tumor growth.

Mesenchymal stem cells are a type of primary cell that self-renew and have multiple differentiating potentials (26). BMMSCs differentiate into nerve cells, skeletal muscle cells, and vascular endothelial cells. Our study also provides evidence that PCa-MSCs have differentiating ability, which is consistent with other reports (14–16,17). Collectively, these findings suggest that in the process of prostate cancer development, MSCs may confer a potential therapeutic advantage against bone metastases in PCa.

One aim of this study was to assess the proliferative activity and growth ability of PCa-MSCs compared with BMMSCs. To achieve this aim, we used cell proliferation and MSC-RM-1 cell culture. The growth curve indicated that the growth ability of PCa-MSCs was markedly higher than that of BMMSCs. In addition, the activity of PCa-MSCs, which could stimulate the cell proliferation of RM-1, was significantly higher when compared with that of BMMSCs. Our results indicate that it may be mediated at least in part by growth factors/chemokines. This observation is consistent with the hypothesis that MSCs locate to the tumor environment since tumors mimic tissue injury (10,11,27). Conversely, MSCs are precursors of stromal cells, which generate the extracellular matrix supporting hematopoiesis within the bone marrow microenvironment (28). Stromal components derived from MSCs may therefore play a role in tumor growth within the tumor microenvironment.

Taking these findings together, it is unlikely that the localization of PCa-MSCs within prostate tumors grown in mice was merely the result of a species-specific tropism. Instead, MSCs appear to have an intrinsic, cell-specific capacity to localize to PCa. Detailed characterization of the properties of MSCs following tumor grafting will be addressed in future studies.
